# True adrenal cyst mimicking renal cancer in a young woman: a case report

**DOI:** 10.4076/1757-1626-2-7351

**Published:** 2009-08-12

**Authors:** Luis Alberto Schlittler, Viviane Weiller Dallagasperina, Jorge Roberto Marcante Carlotto, Rodrigo Ughini Vilarroel, Nícolas Silva Lazaretti

**Affiliations:** Departamento de Oncologia, Hospital da Cidade de Passo FundoRua Tiradentes, 295 - Passo Fundo/RS - Cep 99010-260Brasil

## Abstract

The adrenal cyst is a rare disease that represents approximately 5% of discovered adrenal lesions, which are usually discovered incidentally. True adrenal cysts originate to cells from mesothelium. The potential of cyst adrenal to become malignant has been reported to be 7% and a radical excision of a potentially malignant mass are indicate. We report a case of a 48 year old woman that presented with pain in left hypochondrium and epigastrium, nausea, vomiting, weight loss and microscopic hematuria. After the diagnosis suspicion surgery was performed with a monoblock resection of left kidney and left adrenal gland because of kidney cancer diagnosis as considered. The microscopically analysis of surgical specimen, diagnosed a true epithelial cyst of adrenal gland.

## Introduction

The adrenal cyst is a rare disease. Its incidence in autopsy series varies between 0.064% and 0.18% [1]. The first reported case of adrenal cyst was by Greaseless was in 1670 [2]. Most cases are asymptomatic and the diagnosis is incidental through of Computed Tomography and Magnetic Resonance. There is still no specific radiographic classification criterion for adrenal cysts.

Adrenal epithelial cysts originate to cells from mesothelium that was included in adrenal gland during embryogenesis [3]. When they are symptomatic, symptoms due to compression of adjacent structures may appear and can also simulate cases of renal cysts, splenic cysts, “body” and “tail” of pancreas. His accurate diagnosis is the histological examination.

We report a case of true adrenal cyst, emphasizing its symptoms, differential diagnosis and treatment.

## Case presentation

A 48-year-old Caucasian woman, related pain in left hypochondrium and epigastrium 1 year ago that exacerbated 3 months after the diagnosis, fisgada type that radiates to the back and periumbilical region, associated with symptoms of nausea, vomiting and weight loss (4 kg in 2 months). Myalgia and fatigue were also reported. Personal and Family history had not relevant information’s. On physical examination, abdominal mass was palpable in the left flank.

A urine analysis revealed microscopic hematuria. Abdominal ultrasonography demonstrated a large cystic mass in abdominal upper left quadrant. Computed tomography scans of the abdomen with and without contrast confirmed presence a large cystic lesion, homogenous, well-defined contours, measuring 10.9 × 9.5 cm in largest diameter, in the upper pole of the left kidney and left adrenal without plan between the kidney and adrenal gland.

Based on clinical tests and supplementary examination was considered the diagnosis of kidney cancer and surgery was performed with a monoblock resection of left kidney and left adrenal gland.

**Figure 1.**
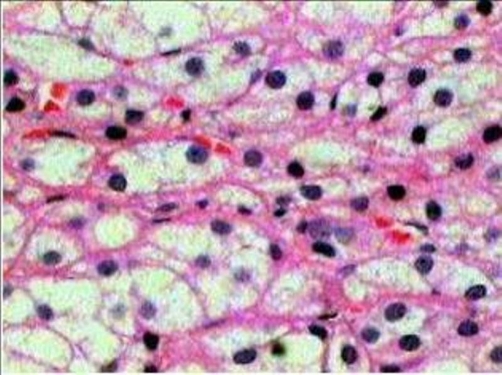
HE 100× cystic lesion covered with single layer of flat cells, without atypia. The wall is made of fibrous tissue with adrenal cortex cellular groups.

**Figure 2.**
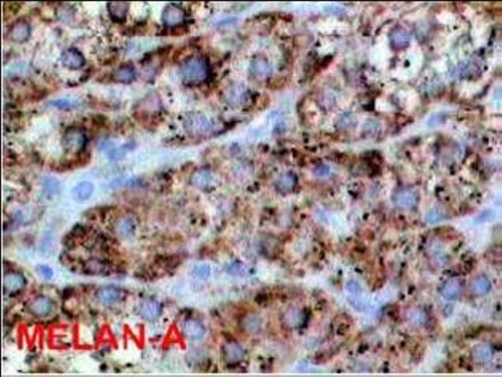
Melan-A positive.

**Figure 3.**
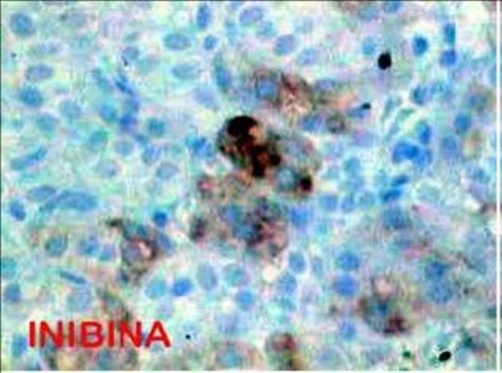
Alpha inhibin positive.

A microscopically analysis of surgical specimen, diagnosed a true epithelial cyst of adrenal gland. Immunohistochemistry demonstrated that the cells lining the cyst were positive for Citoceratina 8, Calretinin, Alpha-inhibin and Sinaptofisina and negative reaction to S-100 protein. A positive reaction for citoceratina 8, alpha-inhibited, melan-A, and calretinin sinaptofisina, confirming presence of adrenal cortex cells (Figures 1-3).

## Discussion

The adrenal glands are very small organs located in the deep retroperitoneal space and are common site of cancer of parasitic disease and inflammatory disease originated in other organs [4].

With the development of advanced image techniques in recent years and growing indications this examination for other purposes, there were increased incidence of adrenal masses detected incidentally. Moreover, it is still not clear if radiological images can distinguish malignant or benign adrenal cysts [4].

The cysts adrenal represent approximately 5% of incidentally discovered adrenal lesions [5,6]. Occasionally, patients complain of flank pain or gastroenteric symptoms. Gastrointestinal symptoms occur when complications occur due to infection, hemorrhage, rupture and compressing adjacent structures [4]. Although adrenal cysts are usually benign, 7% of the lesions are malignant [7].

Rozenblit and colleagues suggested that it is reasonable to evaluate the malignant potential of adrenal cystic lesions by using the Bosniak classification of renal cystic masses. Khoda and associates also used the Bosniak criteria to stratify their patients, and they safely and conservatively managed their patients with uncomplicated adrenal cysts. Even with the Bosniak classification criteria, however, the sensitivity and specificity of definitive differentiating benign and malignant complex cystic adrenal tumors have not been well reported. MRI is thought to be a good diagnostic modality to reveal a variety of tissue characteristics and thus to differentiate the benign or malignant adrenal tumors [8].

A case reported this article, the patient had flank pain, hematuria, and a palpable abdominal mass, triad that are the classic presentation of renal-cell carcinoma [5,7]. Computed tomographic (CT) scan confirmed presence a large cystic lesion, without plan between the kidney and adrenal gland. Whereas approximately 5% of kidney cancers are cystic and lesion was 10.9 cm in largest diameter, the medical assistants opted for realization a monoblock adrenalectomia and nephrectomy respecting the principles of oncology surgery.

Fan et al. observed that fibrous proliferation can eliminate the plane between the kidney and adrenal gland and make radiologic distinction impossible. Indeed, the fibrous capsule surrounding the adrenal gland is known to fuse on occasion with the capsules of the kidneys or liver [9,10].

True adrenal cysts are unusual lesions and usually asymptomatic (Medeiros, 1989). Foster (1966) related, in histopathology, four types of cysts adrenal: endothelial cysts (45%), pseudocysts (39%), epithelial cysts (9%) and parasitic cysts (7%).

Immunohistochemistry analysis of surgical specimen is useful to identify cellular products and markers of cell surface, in case reported, the positive Citoqueratina 8 shows epithelial origin of cyst. The positive immunostains for calretinin and WT-1 lend support to the postulate of Medeiros et al nearly 20 years ago of a mesothelial origin for these cysts [9].

Because of the low incidence of this disease, there were only limited reports of adrenal cyst management in the literature. There is wide acceptance that small, asymptomatic or nonfunctioning adrenal cysts should be managed conservatively. Percutaneous aspiration can be done under guidance of US or CT [2].

Reported indications for operative intervention of adrenal cysts include size (cysts with 6 cm in diameter or more), symptomatic cysts, cysts that secrete excessive amount of hormones, or cysts that are suspicious for malignancy (as determined from imaging studies). For radical excision of a malignant or potentially malignant mass, the principle of surgical oncology - namely, *en bloc* removal of the whole lesion without breaking its integrity before leaving the body - cannot be overemphasized. Potentially malignant or complex adrenal cysts are usually fairly large. Although pure laparoscopic resection of the tumor without violating the cystic wall is not technically impossible, it poses a real challenge to determine how to remove the large tumor intact from the body without making a large skin incision. Laparoscopic adrenalectomy brings many benefits, including smaller incisions, shorter hospital stays, and faster recovery, but open adrenalectomy for adrenal cystic tumors for which there was suspicion of malignancy still accounted for most cases in a review article of adrenal tumors [8].

The treatment is surgical resection of cystic mass, indicated in presence of symptoms, when there are hyperdense components or irregular masses in imaging examinations. It must be accompanied by adrenalectomy when have doubt about the biological behavior of cyst.

Most adrenal cysts are benign and silent clinically, but larger ones up to 30 cm in diameter may require surgical attention [9].

## Abbreviations

CT: computed tomography
US: ultrasonography
WT1: Wilms' tumor suppressor gene
